# Unilateral Biportal Endoscopic Tumor Removal and Percutaneous Stabilization for Extradural Tumors: Technical Case Report and Literature Review

**DOI:** 10.3389/fsurg.2022.863931

**Published:** 2022-03-24

**Authors:** Seung-kook Kim, Riyad Bendardaf, Murtaza Ali, Hyun-a Kim, Eun-jung Heo, Su-chan Lee

**Affiliations:** ^1^Himchan UHS Spine and Joint Center, University Hospital Sharjah, Sharjah, United Arab Emirates; ^2^Joint and Arthritis Research, Orthopedic Surgery, Himchan Hospital, Seoul, South Korea; ^3^Department of Pharmaceutical Medicine and Regulatory Sciences, College of Medicine and Pharmacy, Yonsei University, Incheon, South Korea; ^4^Department of Oncology, University Hospital Sharjah, Sharjah, United Arab Emirates; ^5^Department of Orthopedic Surgery, University Hospital Sharjah, Sharjah, United Arab Emirates

**Keywords:** biportal endoscopic spine surgery, endoscopic spine surgery, spinal cord tumor, tumor biopsy, unilateral biportal endoscopy

## Abstract

**Background:**

Extradural spinal tumors arise from soft or bony tissues in the spine and account for majority of spinal tumors. Interest in the unilateral biportal endoscopic (UBE) technique is rising, because it can easily decompress the bony spinal canal and accommodate all open surgical instruments under endoscopic guidance. However, reports of this technique have been limited to certain diseases. This study first demonstrates the UBE technique for extradural tumor biopsy and removal, and percutaneous stabilization in a 72-year-old female patient with dramatic symptom improvement.

**Methods:**

We used the UBE technique for decompression and the percutaneous screw fixation technique for stabilization in a patient with an extradural mass compressing the thecal sac and destroying the posterior element. Under endoscopic guidance, a unilateral approach was used, and decompression and flavectomy were performed bilaterally. After decompression, tumor removal and biopsy were performed using various forceps and biopsy needles. After confirming sufficient spinal canal decompression, the screw was placed percutaneously. We evaluated the technical process of the procedure, the patient's pre- and postoperative pain (using the visual analog scale), and operative radiology and pathologic results.

**Results:**

Postoperative pain and disability improved clinically, and spinal alignment stabilized radiologically. As the pathology findings confirmed an aneurysmal bone cyst, the treatment was completed without adjuvant therapy.

**Conclusions:**

We treated an unstable spine due to an extradural tumor with the UBE and percutaneous screw techniques.

## Introduction

Extradural spinal tumors arise from soft or bony tissues in the spine, and account for 60% of all spinal tumors (1). Extradural tumors can cause clinical symptoms related to axial destruction of the bony structure, as well as myelopathy and radiculopathy caused by spinal cord and nerve compression (2). To manage this disease entity, physicians should achieve three goals for diagnosis and treatment: pathologic confirmation, neural decompression, and structural reconstruction (3). With the development of endoscopic techniques and instruments, interest in the unilateral biportal endoscopic (UBE) technique is also growing, because it can easily decompress the bony spinal canal and accommodate all open surgical instruments under endoscopic guidance. However, indications and reports of this technique have been limited to degenerative (4) and infectious diseases (5). In this technical note, we describe a step-by-step procedure of how we biopsied the affected tissue and performed tumor removal, spinal canal decompression, and stabilization using the UBE and percutaneous screw placement techniques in a 72-year-old female patient with dramatic symptom improvement.

## Materials and Methods

### Ethics Statement and Case Presentation

**Figure 1:**
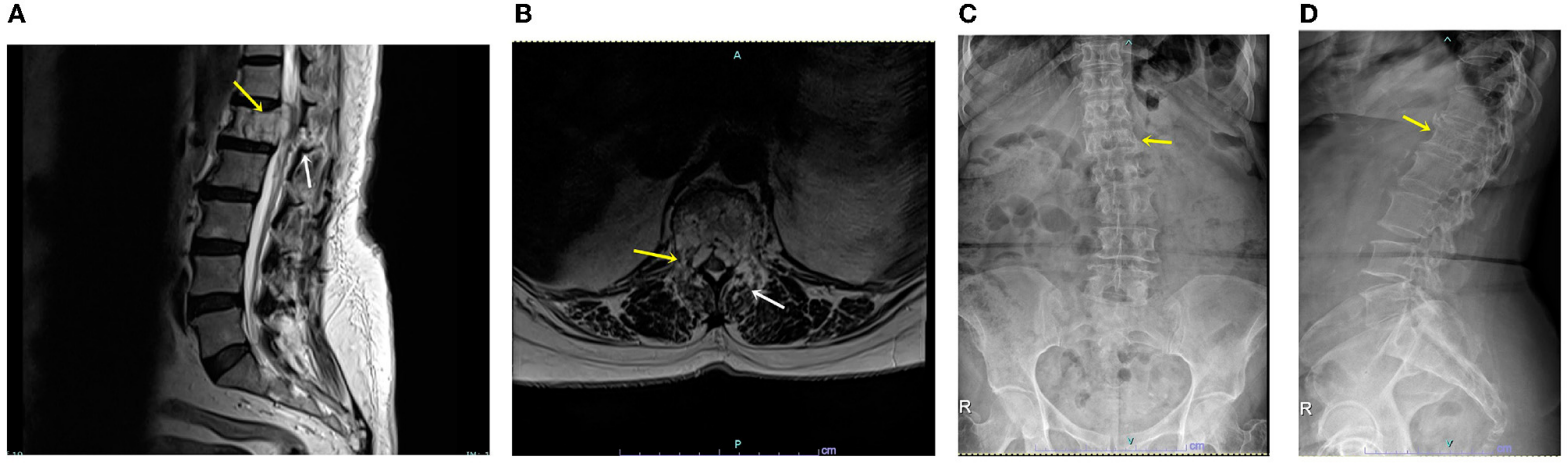
**(A)** Sagittal T2-weighted magnetic resonance image showing a lytic mass (yellow arrow) with spinal canal and posterior involvement (white arrow). **(B)** Axial T2-weighted magnetic resonance image showing the vertebral body, pedicle, and spinal mass (yellow arrow), as well as facet and laminar-mass extension (white arrow). **(C)** Plain anteroposterior radiograph showing decreased vertebral body height (yellow arrow). **(D)** Plain lateral radiograph showing heterogeneous density and decreased vertebral body height (yellow arrow).

We obtained study approval from our institutional review board (approval no.: UHS-HERC-051-10032022), and written consent was obtained from the patient for publication of the report and any accompanying images. The 72-year-old woman visited the outpatient clinic for progressive leg weakness and back pain for the past 3 years. Her back pain was scored as five according to the visual analog scale (VAS), and pain radiating from the buttock to leg was scored as seven on both sides. Bilateral front thigh numbness had started 3 months prior. The patient's knee jerk was 3+, and the hip motor power of both legs decreased subjectively to a grade of four. Preoperative magnetic resonance imaging showed a vertebral body mass with retropulsion into the spinal canal (Figure 1A) and bilateral spinal canal compression caused by an extradural mass (Figure 1B). A homogeneous solitary mass, suspected to be a primary extradural tumor, was noted on radiology. The middle and posterior columns involved the facet and spinous processes. Chest and abdominopelvic computed tomography findings were normal, and blood tumor markers were negative. The Tomita morphological classification (6) was type 4 (extra-compartmental extradural tumor), and the spine instability neoplastic score (7) was 14 (junctional lesion with pain, lysis, bilateral bone collapse, and kyphosis on radiology), indicating an unstable spine. Preoperative radiographs showed decreased vertebral height (Figure 1C) and heterogeneous bone density (Figure 1D). Before the procedure, we explained the possibility of secondary tumor removal if pathology results showed malignancy.

### Procedure

#### Position and Instruments

**Figure 2:**
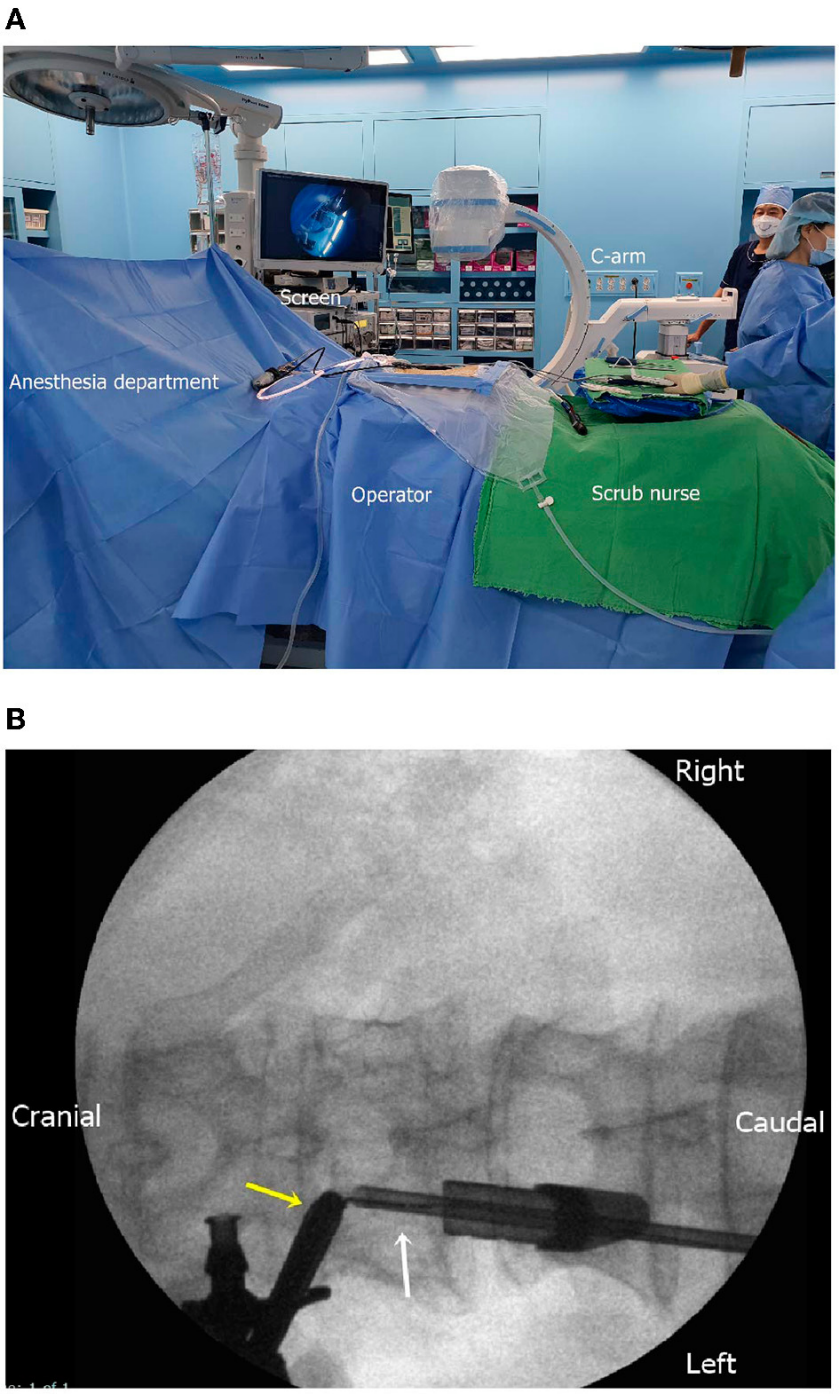
**(A)** Intraoperative setting for biportal endoscopic surgery of the extradural tumor. The C-arm, monitor, continuous irrigation system, and aseptic dressing are prepared. **(B)** Intraoperative fluoroscopic placement of the endoscope (yellow arrow) and instrument (white arrow).

Under general anesthesia, the patient was placed on the spine table (Supplementary Video 1). A surgical drape was placed aseptically, covering the area from the lower thoracic spine to the lumbar spine in a water-tight fashion. For zero-degree endoscopy, a high-definition imaging system, a 3,000-cc sodium chloride irrigation system, and a standard laminectomy set were used (Figure 2A). After the T12–L1 interlaminar space was identified, a scope portal was placed into the interlaminar space cranially, and an instrumental portal was placed caudally (Figure 2B).

### Unilateral Approach and Bilateral Decompression

**Figure 3:**
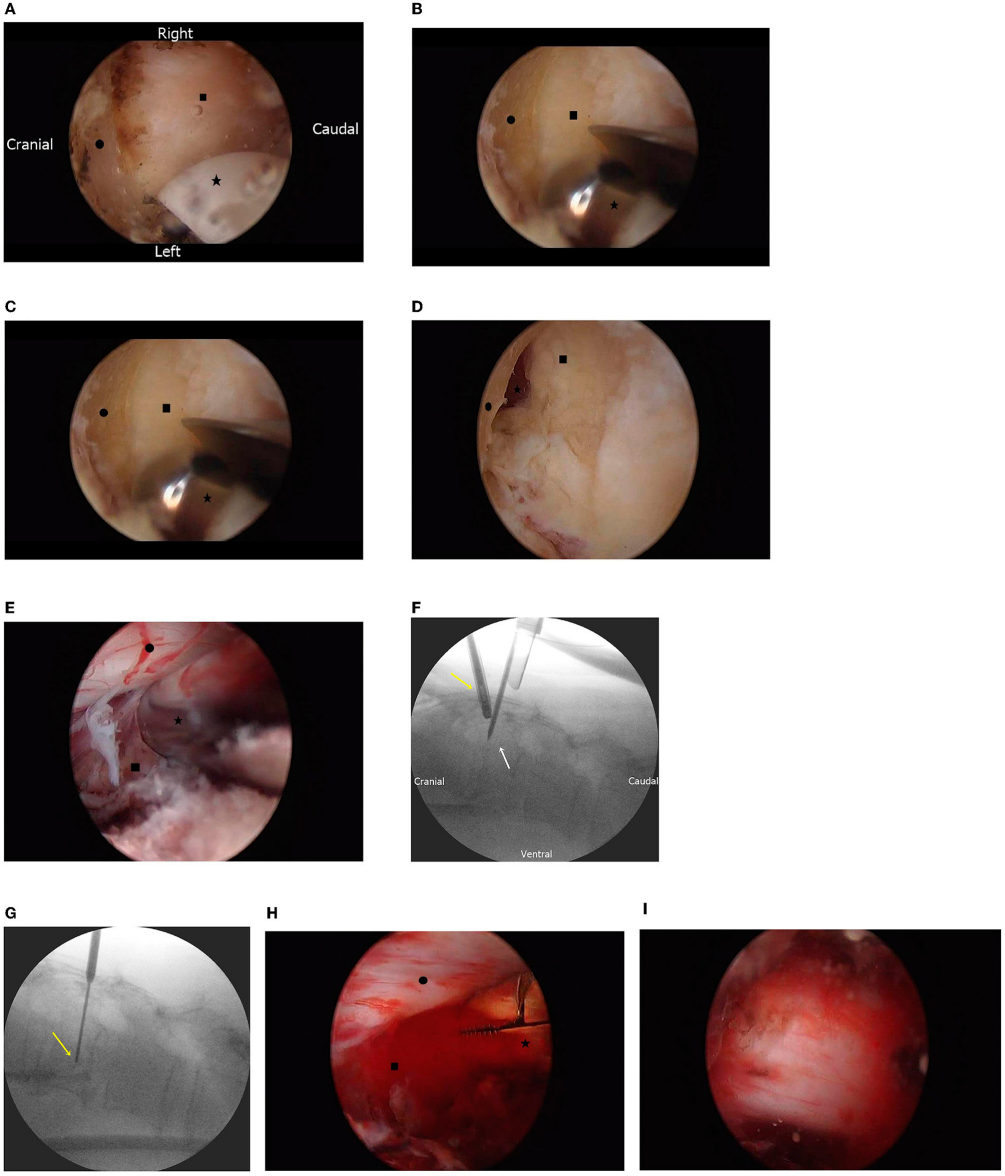
**(A)** Endoscopic findings of the prepared working space. The lower border of the lamina (•) and interlaminar space (■) are identified using the radiofrequency electrode (⋆). **(B)** Endoscopic findings of laminectomy. The upper lamina (•) and ligamentum flavum (■) are identified, and bone is drilled with an automated drill (⋆). **(C)** Endoscopic findings of flavectomy. Under the middle of the partially removed lamina (•), both the ligamentum flava (■) and epidural space are identified (⋆) under the top of the ligamentum flavum. **(D)** Endoscopic findings after removal of the ligamentum flavum. Under the thecal sac and nerve root (•), the hypervascular mass is identified (■). **(E)** Endoscopic-guided needle biopsy. By needle biopsy (⋆), the tumor (■) tissue is obtained without causing nerve injury (•). (F) Fluoroscopic image of the endoscopic-guided tumor biopsy. Under endoscopic guidance (yellow arrow), the biopsy needle is inserted (white arrow). **(G)** Fluoroscopic image of the needle biopsy. The biopsy needle (yellow arrow) is adjusted; the amount of tissue obtained is dependent on vertebral body depth. **(H)** Endoscopic finding of tumor removal with an instrument. The vascular mass (■) is removed with pituitary forceps (⋆) without causing nerve injury (•). **(I)** After tumor removal and bilateral decompression, the spine is decompressed and pulsated.

Using a radiofrequency coagulator, we identified the lower end of the upper lamina and interlaminar space as landmarks for laminectomy (Figure 3A). Laminectomy was performed using an automated drill (Figure 3B), and bilateral flavectomy was performed using pituitary forceps and Kerrison punches (Figure 3C). After bilateral interlaminar decompression was performed, the thecal sac and extradural mass under the pedicle were identified (Figure 3D).

#### Tumor Biopsy and Removal

We performed needle biopsy under endoscopic guidance (Figures 3E,F). The depth and location of the tumor were confirmed by fluoroscopy, and a tissue was obtained (Figure 3G). The tumor mass was removed with pituitary forceps and a curette (Figure 3H), and it was suspected that hemostasis was required. Bilateral thecal sac decompression was performed after tumor removal and bleeding control (Figure 3I).

#### Percutaneous Screw Fixation

**Figure 4:**
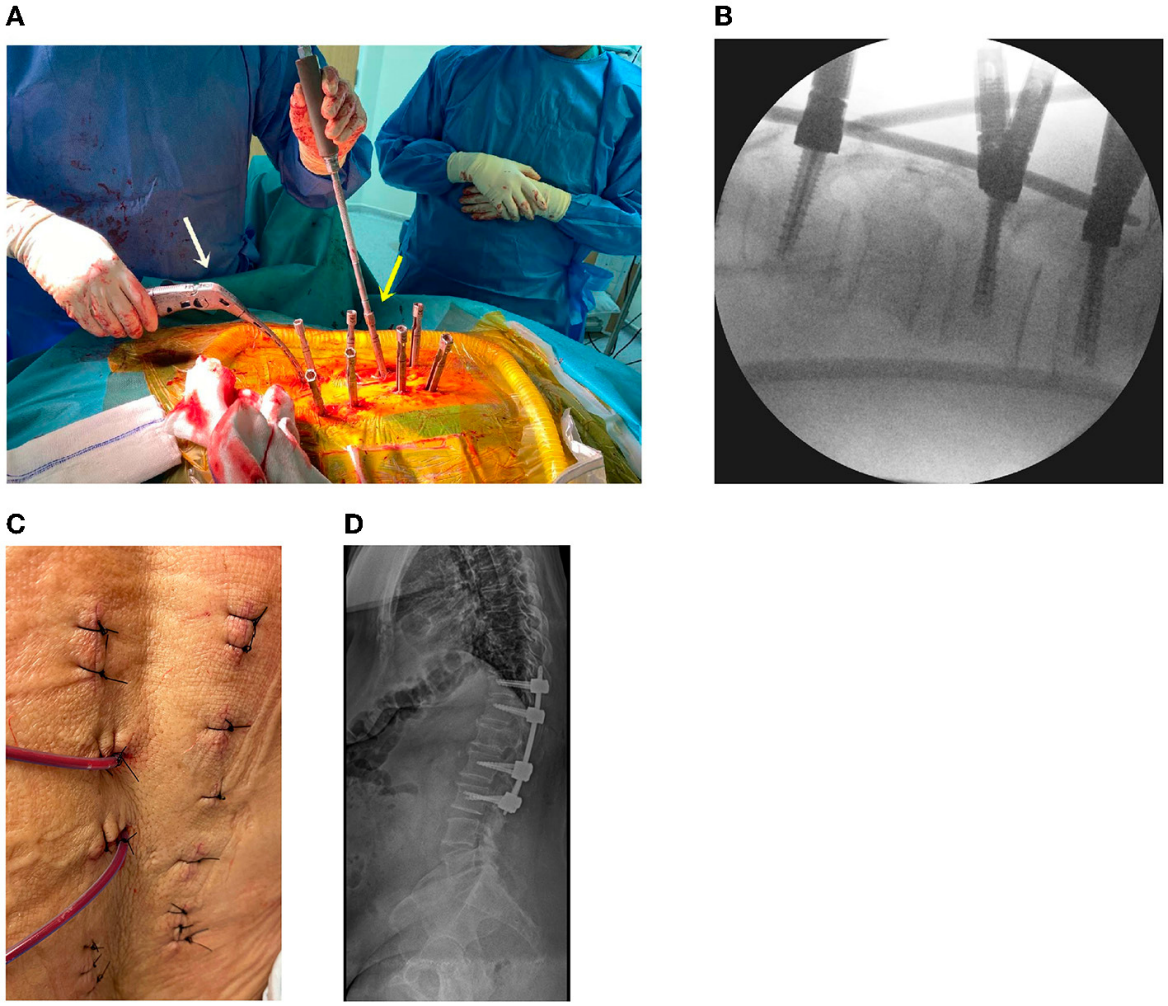
**(A)** Operative setting using the percutaneous screw fixation procedure. Screw capping (yellow arrow) and rod placement (white arrow) are performed. **(B)** Intraoperative fluoroscopy for screw and rod application. **(C)** The skin is sutured after the surgery. **(D)** Plain radiograph after the surgery.

Using a previous endoscopic incision, an instrumental portal, and additional incisions, percutaneous pedicle screws were inserted into T11, T12, L2, and L3 using a percutaneous screw system (CD Horizon Solera Voyager Spinal System; Medtronic, Memphis, TN, United States). After bilateral screw placement, the rod was connected bilaterally (Figures 4A,B). A drainage bag was inserted into the tumor removal site, and the skin was sutured with 3-0 nylon (Figure 4C). After all procedures were completed, radiography was performed to confirm stabilization (Figure 4D). Postoperatively, pain was managed with acetaminophen (100 mg, thrice daily, intravenous), and third-generation cephalosporin antibiotics were administered for 3 days.

## Results

**Figure 5:**
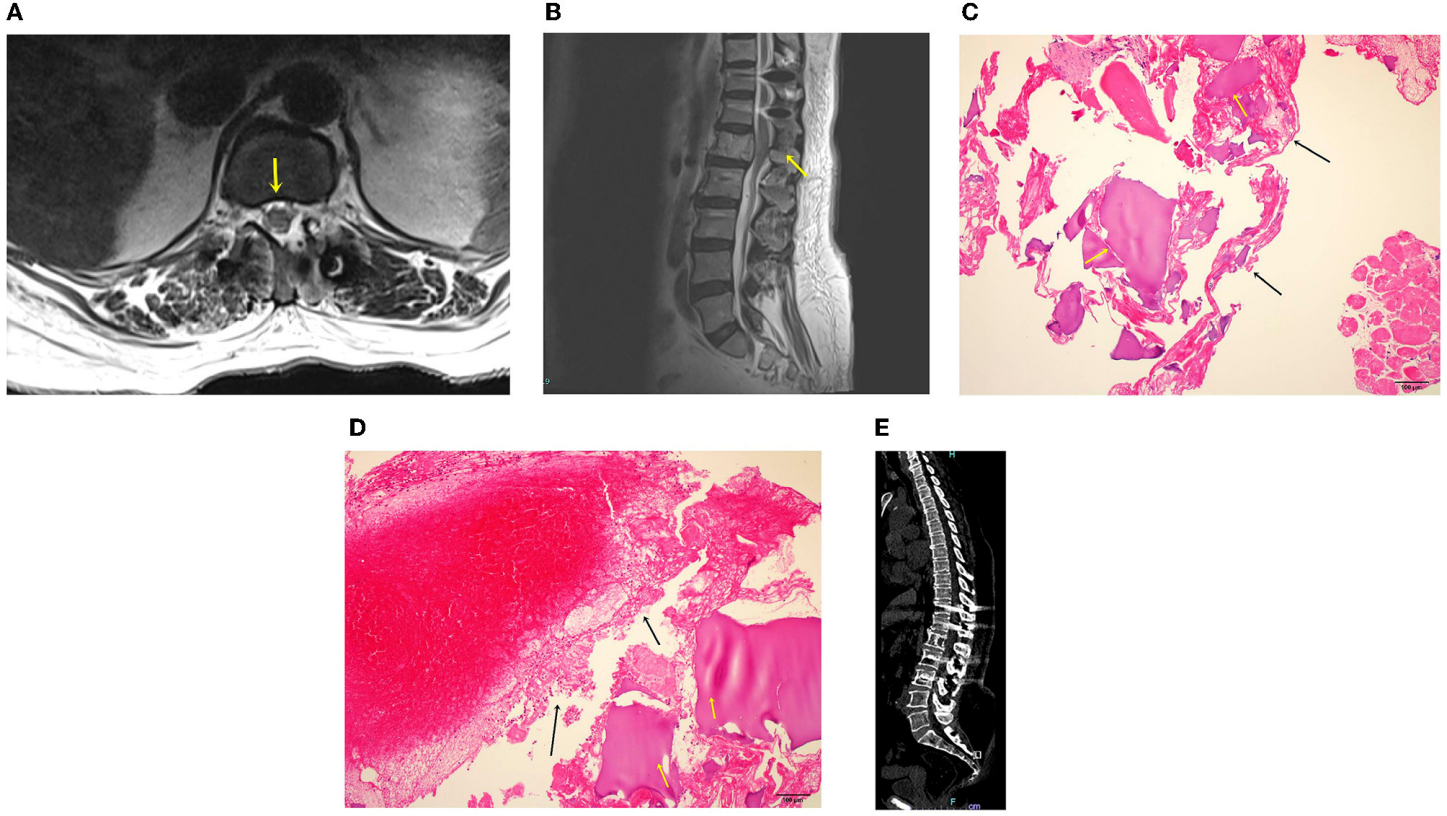
**(A)** Magnetic resonance image after bilateral decompression. Spinal canal widening is complete, and the thecal sac is decompressed (yellow arrow). **(B)** Sagittal magnetic resonance image after the procedure. The spinal canal is decompressed, and the screw is placed. **(C)** Hematoxylin and eosin (H & E) staining of the surgical biopsy specimen. A chondroid material (black arrows) and a blood clot (yellow arrows) are identified. **(D)** H & E staining of the surgical biopsy specimen. The chondroid material (black arrows) and blood clot (yellow arrows) are shown. The 12-month postoperative computed tomography showed no more extension and removed tumor **(E)**.

After the surgery, imaging showed that the thecal sac was decompressed bilaterally (Figure 5A), and that the retropulsed tumor was subtotally removed (Figure 5B). The patient's bilateral leg numbness improved to a VAS score of 1, and weakness improved on postoperative day 1. The biopsy results showed chondroid material with a blood clot, and the final diagnosis was aneurysmal bone cyst (Figures 5C,D). Following the oncologist's opinion, treatment was completed without adjuvant radiotherapy or chemotherapy. Follow-up radiographs were obtained after 1, 3, 6, and 12 months, and computed tomography (Figure 5E) and magnetic resonance imaging were performed 12 months postoperatively. At the 12-month follow-up, the tumor had not recurred, the spinal alignment was stable, and the patient was asymptomatic.

## Discussion

To the best of our knowledge, this study was the first to apply the UBE and percutaneous screw fixation techniques for extradural tumor treatment. Our goals were pathologic confirmation, spinal cord decompression, and stabilization of the spinal column; all of which were achieved. The patient was satisfied with her dramatically improved clinical symptoms.

A solitary spinal mass with or without symptoms is usually suspected as a primary spinal tumor or metastasis from another organ. Primary non-lymphoproliferative spinal tumors account for <5% of all bony tumors; therefore, spinal metastases are more frequent [60% of all spinal tumors (8)] than primary masses. However, pathologic confirmation is necessary for a solitary mass in the spine without symptoms related to other organs, and surgical treatment should be chosen based on clinical and radiologic findings.

Fine-needle biopsy is recommended for pathologic confirmation when diagnosing a solitary spinal mass without instability (9). However, for a symptomatic spinal canal mass, treatment should be based on clinical symptoms and radiologic instability. For a solitary mass with possible malignancy, total removal with *en bloc* resection is recommended (10). Because of the invasiveness of this operation, other surgical options such as subtotal resection, intracystic injection, and embolization are recommended in cases of benign pathology, especially for aneurysmal bone cysts (11). Intracystic injection and embolization cannot decompress the spinal canal and may lead to leakage, and symptoms can consequently worsen. Therefore, less invasive procedures with spinal decompression should be pursued for benign masses involving the spinal canal.

The literature on the biportal technique is still limited to treatments for degenerative disease entities, including spinal stenosis decompression, herniated disc removal, and interbody fusion for instability (12). The advantages of the biportal technique compared with other techniques include shorter hospital stay and less postoperative back pain based on preservation of the back muscle (13). This technique has strengths in bilateral decompressive laminotomy and flavectomy, because free movement of the scope is easy and possible on the contralateral side of the thecal sac. Compared with the uniportal technique, the biportal technique allows for insertion of various surgical instruments without limitations of the cannula, and bone and tissue removal is easy. With extradural tumors, the UBE technique allows for decompression of the spinal canal with various surgical instruments and safe removal of a tumor by utilizing a high-definition imaging system.

Percutaneous screw stabilization for metastatic spinal tumors involves a short operative time, minor intraoperative bleeding, and a short hospital stay (14). Without muscle dissection, accurate placement of the pedicular screw is possible with a small incision. A recent system can insert rods without needing additional incisions; therefore, this technique has become easier. Placement is not limited to tumors and areas of trauma in degenerative spinal diseases (15). Extradural tumors or trauma injuries with spinal cord compression are possible indications for this procedure. Hospital stay was only 2 days with both endoscopic techniques, and postoperative opioids were not administered. Accordingly, medical costs can also decrease. However, there are reports of percutaneous screw fixation without fusion material showing low fusion rate compared with the fusion technique (16). Therefore, indications for percutaneous screw fixation should be considered elderly and short level involvement only (17).

There are certain points that surgeons should consider. The incision should be wider than that in degenerative surgery. The authors recommend that each hole be 1 cm and that the rod insertion site be at least 2 cm. A relatively larger incision can help surgeons identify bleeding and allow for more efficient tumor removal and screw and rod insertion. Unclear vision can prolong the operation time and water retention in the soft tissue. If bleeding occurs during the procedure, bone bleeding should be controlled using an RF coagulator or bone wax. If bleeding focus is not clear, prothrombin hemostatic matrix and compression are useful options for solving the problem (18).

Our technique has some limitations in terms of its broader application. First, a mass suspected to be malignant in radiology should be totally removed. Second, the endoscopic technique cannot expose a wide range like the microscopic technique, and tissues can be lost due to continuous water irrigation. More case evaluations involving this technique are needed, even with its limited indications. Additionally, prospective, multicenter case studies are essential for evaluating outcomes associated with this technique. With the continuous development of new techniques and comparisons with other techniques, it is necessary to evaluate outcomes further.

In conclusion, we described a biportal technique for spinal canal decompression, tumor removal, and biopsy, as well as a percutaneous stabilization technique. With the development of instruments and surgical techniques, our combined technique will play a role in spinal oncology.

## Data Availability Statement

The original contributions presented in the study are included in the article/Supplementary material, further inquiries can be directed to the corresponding author.

## Ethics Statement

The studies involving human participants were reviewed and approved by University Hospital Sharjah Institutional Review Board. The patients/participants provided their written informed consent to participate in this study. Written informed consent was obtained from the individual(s) for the publication of any potentially identifiable images or data included in this article.

## Author Contributions

S-kK: conceptualization, resources, writing (original draft preparation), writing (review and editing), visualization, and project administration. H-aK: methodology. E-jH: software and investigation. RB, S-kK, and S-cL: validation. MA: formal analysis. S-cL: data curation and supervision. All authors have read and agreed to the published version of the manuscript. All authors contributed to the article and approved the submitted version.

## Conflict of Interest

The authors declare that the research was conducted in the absence of any commercial or financial relationships that could be construed as a potential conflict of interest.

## Publisher's Note

All claims expressed in this article are solely those of the authors and do not necessarily represent those of their affiliated organizations, or those of the publisher, the editors and the reviewers. Any product that may be evaluated in this article, or claim that may be made by its manufacturer, is not guaranteed or endorsed by the publisher.

## Supplementary Material

Supplementary Video 1 The entire operative procedure including endoscopic tumor removal, tumor biopsy, and percutaneous screw fixation.

## Abbreviations

H & E: hematoxylin and eosin
UBE: unilateral biportal endoscopic
VAS: visual analog scale.
